# Quantitative analysis of colon perfusion pattern using indocyanine green (ICG) angiography in laparoscopic colorectal surgery

**DOI:** 10.1007/s00464-018-6439-y

**Published:** 2018-09-10

**Authors:** Gyung Mo Son, Myeong Sook Kwon, Yoonhong Kim, Jisu Kim, Seung Hwa Kim, Jung Woo Lee

**Affiliations:** 1 Department of Surgery, Pusan National University Yangsan Hospital, Yangsan, Gyeongsangnam-do 50612 Republic of Korea; 2 Medical Research Institute, Pusan National University, Busan, Republic of Korea; 3 Department of Medicine, School of Medicine, Pusan National University, Yangsan, Gyeongsangnam-do Republic of Korea; 4 Department of Medical Image Analysis, Withpia.co, Busan, Republic of Korea

**Keywords:** Indocyanine green, Anastomotic complications, Laparoscopy, Colorectal surgery, Intraoperative angiography, Quantitative analysis

## Abstract

**Purpose:**

This study aimed to quantitatively evaluate colon perfusion patterns using indocyanine green (ICG) angiography to find the most reliable predictive factor of anastomotic complications after laparoscopic colorectal surgery.

**Methods:**

Laparoscopic fluorescence imaging was applied to colorectal cancer patients (*n* = 86) from July 2015 to December 2017. ICG (0.25 mg/kg) was slowly injected into peripheral blood vessels, and the fluorescence intensity of colonic flow was measured sequentially, producing perfusion graphs using a video analysis and modeling tool. Colon perfusion patterns were categorized as either fast, moderate, or slow based on their fluorescence slope, *T*_1/2MAX_ and time ratio (TR = *T*_1/2MAX_/*T*_MAX_). Clinical factors and quantitative perfusion factors were analyzed to identify predictors for anastomotic complications.

**Results:**

The mean age of patients was 65.4 years, and the male-to-female ratio was 63:23. Their operations were laparoscopic low anterior resection (55 cases) and anterior resection (31 cases). The incidence of anastomotic complication was 7%, including colonic necrosis (*n* = 1), anastomotic leak (*n* = 3), delayed pelvic abscess (*n* = 1), and delayed anastomotic dehiscence (*n* = 1). Based on quantitative analysis, the fluorescence slope, *T*_1/2MAX_, and TR were related with anastomotic complications. The cut-off value of TR to categorize the perfusion pattern was determined to be 0.6, as shown by ROC curve analysis (AUC 0.929, *P* < 0.001). Slow perfusion (TR > 0.6) was independent factor for anastomotic complications in a logistic regression model (OR 130.84; 95% CI 6.45–2654.75; *P* = 0.002). Anastomotic complications were significantly correlated with the novel factor TR (> 0.6) as the most reliable predictor of perfusion and anastomotic complications.

**Conclusions:**

Quantitative analysis of ICG perfusion patterns using *T*_1/2MAX_ and TR can be applied to detect segments with poor perfusion, thereby reducing anastomotic complications during laparoscopic colorectal surgery.

Colon cancer is one of the most common gastrointestinal cancers worldwide. The incidence of colorectal cancer has been increasing year by year, and oncologic outcomes have been improved by the development of surgical techniques and chemotherapy regimens in Korea. However, despite the improvement of surgical techniques and postoperative care, anastomotic complications still occur at a rate as high as 10 ~ 20% [1–4]. It is known that 10% of the population has poor development of the collateral circulation branches around the splenic flexure of the colon, and these differences in the vascular anatomical structure can lead to colonic ischemia in some patients after colorectal surgery [2–7]. Acute ischemia at the anastomosis can lead to anastomotic leakage or colon necrosis [8]. Chronic ischemia may cause anastomotic stricture, requiring reoperation [9, 10].

The most commonly used method for evaluating colonic perfusion is for the surgeon to observe the color change or pulsation of small blood vessels on the colon wall with their own eyes [11–13]. Sometimes, it is not easy to accurately detect minute changes in the microcirculation of the colon wall by visual observation. Especially when blood vessels are buried in fat tissue due to visceral obesity, even experienced surgeons can have significantly reduced accuracy in assessing colonic perfusion [12, 14].

The recently developed fluorescence camera system using indocyanine green (ICG) can be used to easily observe the perfusion status of the colon during robotic or laparoscopic surgery. In previous studies, the effects of using ICG fluorescence to protect from anastomotic complications have not been clearly demonstrated [15–18]. There is a limit to predicting colon ischemia by only qualitatively evaluating ICG fluorescence expression. Even if ICG fluorescence is expressed in the large intestine, colonic ischemia may occur if the blood flow rate is lower than the physiological requirements. Reliable quantitative analysis for predicting anastomotic complications is still not sufficient, and until now there have been many limitations in clinically applying the quantitative analysis of ICG fluorescence in the surgical field [19]. Recently, research on the quantitative analysis of colonic perfusion status has been continued in some institutions [20–23].

The goal of this study is to quantitatively evaluate colon perfusion patterns using ICG angiography to find the most reliable predictive factor of anastomotic complications after laparoscopic colorectal surgery.

## Materials and methods

### Patients

This study was performed on patients who underwent laparoscopic surgery for colorectal cancer from July 2015 to December 2017 at the Yangsan Pusan National University Hospital. The inclusion criteria were patients who were 18 ~ 85 years old, had sigmoid or rectal cancer, and who underwent anterior or low anterior resection (LAR) with primary anastomosis with or without diverting ileostomy. The exclusion criteria were hemodynamic instability, emergent surgery, and pregnancy. To reduce the risk of cross-reactivity, all patients participating in the study were found to have no history of allergies or adverse reactions to either the contrast agent for computed tomography (CT) or drugs containing iodine. This study was conducted after receiving the approval of the Institutional Review Board (IRB No. 05-2016-169) of the Pusan National University Yangsan Hospital. Written informed consent was obtained from all patients included in this study.

### ICG angiography

**Fig. 1:**
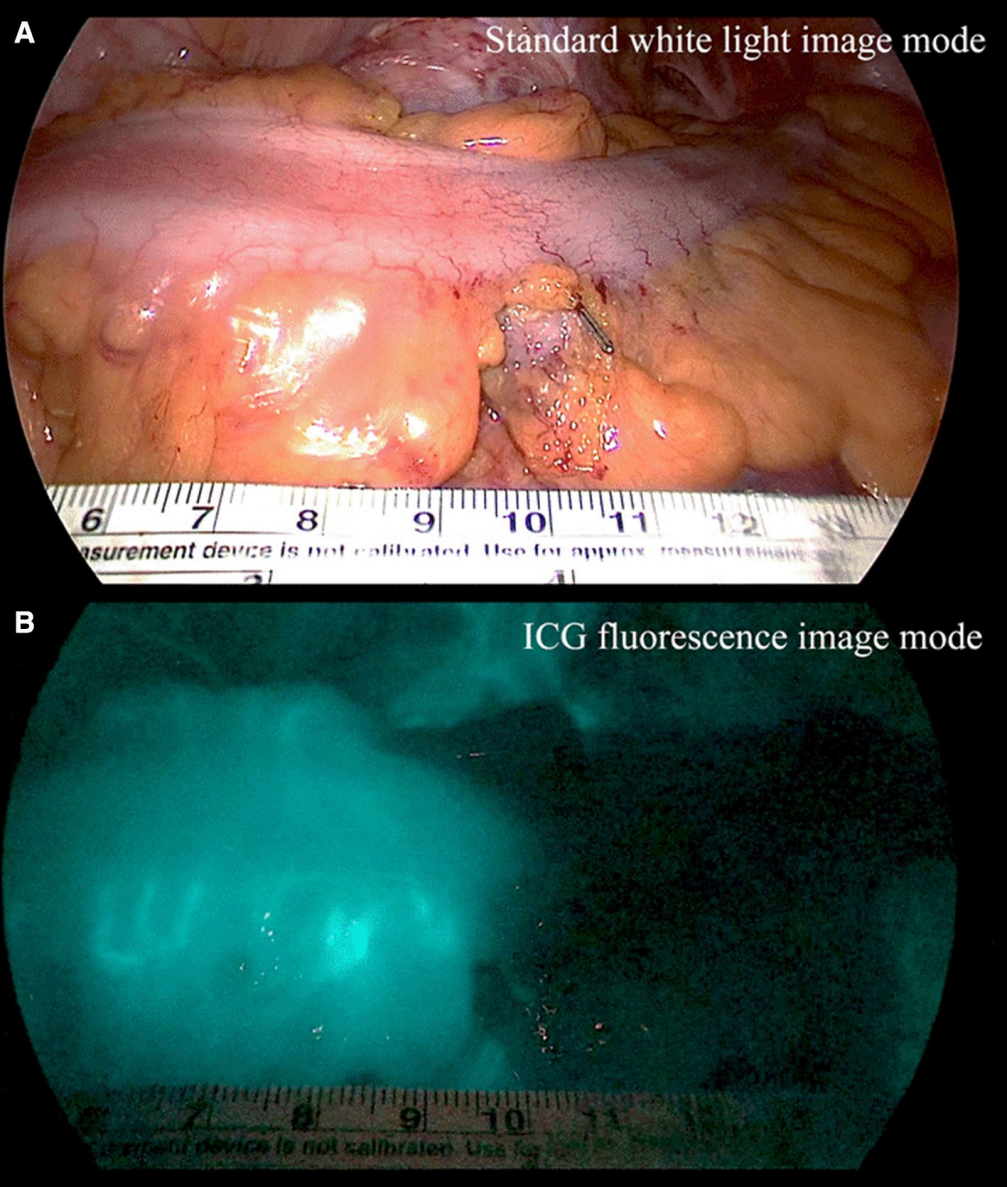
Sigmoid colon image under standard white light (**A**) and ICG fluorescence with red inversion mode (**B**) during laparoscopic low anterior resection for a rectal cancer patient

In laparoscopic colorectal surgery, the inferior mesenteric artery was ligated at a high level (high ligation) or a low level with left colic artery preservation (low ligation). Then, the upper colon was mobilized and the proximal colon transection was prepared by mesenteric division. ICG angiography was performed with a fluorescence imaging system (IMAGE1 S™, Karl Storz, Germany). ICG (25 mg, Daiichi Sankyo, Tokyo, JP) was diluted in 10 ml of distilled water, and a minimum dose of 0.25 mg/kg was slowly injected into the peripheral blood vessels for 10 s. Intravenous ICG then bound to intravascular globulin or albumin and remained in the vascular circulation. Then, a near infrared ray with a wavelength of 800 nm was emitted from the laparoscopic camera, causing ICG in the blood vessel to emit a wavelength of 803 nm, and the fluorescence image was outputted to the monitor. Colonic blood perfusion was observed for 2 min after ICG injection, and the colon was observed for up to 5 min if enough blood flow was not observed within 2 min. In the early period, the colon was extracted from the abdominal cavity through the mini-laparotomy site, and the fluorescence intensity was measured using standard ICG mode. After the midpoint of this study, colon fluorescence images were obtained inside the abdominal cavity, under red inversion mode (Fig. 1).

### Quantitative analysis of colonic perfusion

**Fig. 2:**
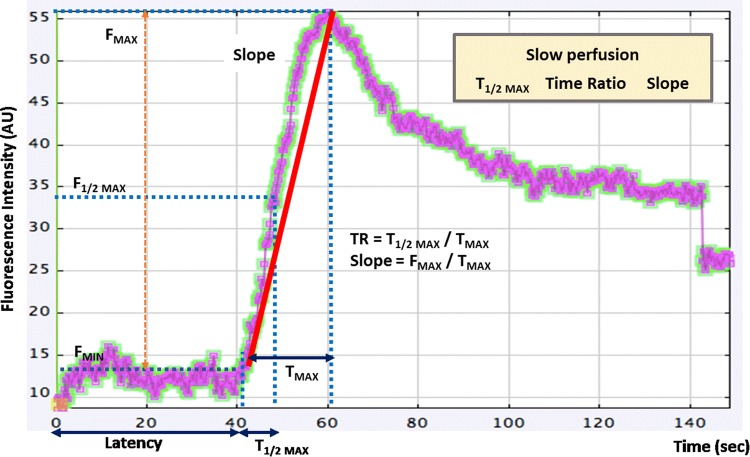
Time–fluorescence curve of ICG angiography for quantitative perfusion analysis. *F* fluorescence intensity, *TR* time ratio. The red line represents the slope (Δ*F*/Δ*T*) of the fluorescence intensity. Slow perfusion was associated with *T*_1/2MAX_, TR and slope

Colonic fluorescence videos were recorded, and the change in fluorescence intensity was measured sequentially to produce colonic perfusion graphs using a video analysis and modeling tool (Tracker 4.97, Douglas Brown, Open Source Physics, Boston MA, USA) [24]. To quantitatively assess the colonic perfusion, fluorescence intensity factors and perfusion time factors were calculated from each ICG fluorescence graph (Fig. 2). The fluorescence intensity factors were fluorescence intensity at baseline (*F*_MIN_), fluorescence difference between maximum and baseline intensity (*F*_MAX,_ Δ*F*), and the fluorescence slope (Slope = Δ*F*/Δ*T* = *F*_MAX_/*T*_MAX_). The perfusion time factors were the time from first fluorescence increase to maximum (*T*_MAX_ = Δ*T*), time from first fluorescence increase to half of maximum (*T*_1/2MAX_), and the time ratio (TR = *T*_1/2MAX_/*T*_MAX_).

### Effect evaluation

We investigated the predictive factors of anastomotic complications by analyzing related clinical factors and perfusion factors to assess the efficacy of ICG reperfusion studies. The primary outcome was the occurrence of anastomotic complications requiring invasive treatment like ileostomy or percutaneous drainage. Anastomotic complications were defined as leakage, colonic necrosis, pelvic abscess, and stricture requiring endoscopic dilatation or surgical treatment. If postoperative anastomotic complications were suspected within 30 days after surgery, pelvic CT or sigmoidoscopy were performed to evaluate short-term anastomotic complications. To evaluate long-term complications, pelvic CT, sigmoidoscopy, or colonoscopy were performed either six months or one year after the operation to check for anastomotic stenosis or sinus formation.

We used a prospectively recorded database to identify clinical factors related to anastomotic complications like age, gender, body mass index (BMI), American Society of Anesthesia (ASA) score, serum albumin, hypertension, diabetes, smoking, serum total cholesterol, pathologic T and N status, preoperative concurrent chemoradiation therapy (CCRTx), cancer obstruction, operation name, anastomosis level, number of linear cutter staples used for rectal transection, splenic flexure mobilization, ileostomy, and the ligation level of the inferior mesenteric artery (IMA).

### Statistics

Chi-square and Pearson correlation tests were used to evaluate the correlation between clinical factors and anastomotic complications. We used two-sided independent samples *t* tests for continuous variables of perfusion factors to identify risk factors of anastomotic complications.

Receiver operating characteristic (ROC) curves were used to determine the cut-off values of perfusion factors with high sensitivity and specificity. The perfusion status was divided into two groups according to the criteria, and the incidence of anastomotic complications was compared to evaluate diagnostic values. Curve estimation regression analysis with a logarithmic model was used to evaluate the correlations between perfusion factors and to establish a step-by-step flow chart to predict anastomotic complications. To evaluate the predictive effect of perfusion factors for anastomotic complications, multivariate analysis was performed using logistic regression analysis. The covariance input criterion was < 0.1 and the elimination criterion was < 0.05. SPSS 24.0 (Statistical Package for Social Science Version 24.0, IBM SPSS, Armonk, NY, IBM Corp) was used for statistical analyses, and the significance level was p-value less than 0.05.

## Results

### Characteristics of patients

The mean age of the patients was 65.4 years, and the male-to-female ratio was 63:23. Laparoscopic low anterior resection (55 cases) and laparoscopic anterior resection (31 cases) were performed. Six of the 86 (7%) patients with colorectal cancer had major anastomotic complications requiring invasive treatment. The anastomotic complications were colonic necrosis (*n* = 1), anastomotic leak (*n* = 3), delayed pelvic abscess (*n* = 1), and delayed anastomotic dehiscence during intensive care for respiratory failure (*n* = 1). Reoperation was required in three cases (3.5%). Total colectomy for colon necrosis (*n* = 1) and diverting ileostomy (*n* = 2) for anastomotic leak were performed. In two patients who underwent ileostomy at the first operation, pelvic abscess with delayed anastomotic leak was found, and percutaneous catheter drainage was performed. Anastomotic stricture (*n* = 1), through which it was difficult to pass the endoscope, was not definitely observed in defecography and improved after restoration of ileostomy without any treatment. In the complication group, the rate of smoking and low anastomosis level (< 5 cm) were higher than among patients with no complications, and there were significantly fewer splenic flexure mobilizations. The proportion of patients with low BMI (< 25 kg/m^2^), LAR, and linear cutter staple usage (≥ 2) showed an elevated trend in the complication group (Table 1).

**Table 1:** Characteristics of patients (*n* = 86)

Clinical factor	Anastomotic complications		*P* value
	(−) (*n* = 80)	(+) (*n* = 6)	
Age (≥ 70 years)	34 (42.5%)	1 (16.7%)	0.214
Male:Female	57:23	6:0	0.125
BMI (≥ 25 kg/m^2^)	30 (37.5%)	0 (0%)	0.063
ASA score (≥ III)	8 (10.0%)	1 (16.7%)	0.607
Albumin (< 4 g/dL)	13 (16.3%)	1 (16.7%)	0.979
Hypertension	36 (45%)	2 (33.3%)	0.579
Diabetes	18 (22.5%)	3 (50%)	0.130
Smoking (> 40 pack-year)	22 (27.5%)	4 (66.7%)	0.044
Cholesterol (≥ 220 mg/dl)	8 (10.7%)	1 (16.7%)	0.653
T status 3–4	54 (67.5%)	6 (100%)	0.095
N status 1–2	34 (42.5%)	2 (33.3%)	0.661
Preoperative CCRTx	3 (3.8%)	1 (16.7%)	0.147
Cancer obstruction	19 (23.8%)	3 (50%)	0.155
Operation (LAR)	49 (61.3%)	6 (100%)	0.057
Anastomosis level (< 5 cm)	24 (30%)	5 (83.3%)	0.008
linear cutter stapler (≥ 2)	23 (28.8%)	4 (66.7%)	0.054
SFM	61 (76.3%)	2 (33.3%)	0.022
Ileostomy	28 (35%)	3 (50%)	0.460
IMA (high ligation)	59 (73.8%)	6 (100%)	0.149
Transection line change	6 (7.5%)	1 (16.7%)	0.428

*BMI* body mass index, *ASA* American society of anesthesia, *CCRTx* concurrent chemoradiation therapy, *LAR* low anterior resection, *SFM* splenic flexure mobilization, *IMA* inferior mesenteric artery

In the complication group, the fluorescence factor tended to show relatively lower intensity, and the fluorescence slope was significantly lower. Finally, the perfusion time-related factors were significantly delayed in the complication group (Table 2).

**Table 2:** Correlation between perfusion factors and anastomotic complications

Perfusion factor	Parameters	Anastomotic complications		*P* value
		(−) (*n* = 80)	(+) (*n* = 6)	
Fluorescence intensity-related factors	*F*_MIN_ (AU)	10.6 ± 1.0	11.5 ± 2.5	0.820
	*F*_MAX_ (AU)	58.0 ± 3.4	34.9 ± 7.4	0.074
	Slope (AU/sec)	2.5 ± 0.2	0.7 ± 0.2	< 0.001
Perfusion time-related factors	*T*_MAX_ (sec)	30.3 ± 2.3	64.0 ± 11.7	< 0.001
	*T*_1/2MAX_ (sec)	11.7 ± 0.8	40.37 ± 7.8	< 0.001
	TR	0.4 ± 0.0	0.6 ± 0.0	< 0.001

*P* value (two-sided independent samples *t* test). The values are expressed as mean ± standard error

*F*_*MIN*_ fluorescence intensity on baseline, *F*_MAX_ fluorescence difference between maximum and baseline intensity, *Slope* (Slope = ∆*F*/∆*T* = *F*_MAX_/*T*_MAX_), *T*_MAX_ time from first fluorescence increase to maximum (∆*T*), *T*_*1/2MAX*_ time from first fluorescence increase to half of maximum, *TR* time ratio (TR = *T*_1/2MAX_/*T*_MAX_), *AU* arbitrary unit

ROC analysis was performed to obtain an appropriate cut-off value for the perfusion factors predicting anastomotic complications. The area under curve (AUC) of *T*_1/2MAX_ and TR were higher than 0.9 and showed significant predictive value for anastomotic complications. The fluorescence slope was correlated with anastomotic complication, but its low AUC showed that it is limited as a predictive index (Table 3).

**Table 3:** Receiver operating characteristic (ROC) curve analysis (*n* = 86)

Perfusion factor	Cut-off value	AUC	*P* value	95% CI
Slope (AU/sec)	0.7	0.123	0.002	0.001–0.245
*T*_1/2MAX_ (sec)	18	0.963	< 0.001	0.910–1.0
TR	0.6	0.929	< 0.001	0.845–1.000

*Slope* (fast > 1.0 AU/sec, slow < 0.7 AU/sec), *T*_1/2MAX_ (fast < 10 s, slow > 18 s), *TR* time ratio (fast < 0.4, slow > 0.6), *AUC* area under curve, *AU* arbitrary unit, *CI* confidence interval

The perfusion status of patients with anastomotic complications was classified using the cut-off value obtained from the ROC curve. Anastomotic complications were mainly distributed among patients with slow perfusion, shown by low values of *T*_1/2MAX_ and TR; such values represent a dangerous zone. Fast perfusion leads to minimal risk of complications, and we estimate it to be a safe zone (Table 4).

**Table 4:** Classifications of risk zone for predicting anastomotic complications (*n* = 86)

Risk zone	Perfusion status	Slope*	*T*_1/2MAX_ *	TR*
Safe zone	Fast	1/60 (1.7%)	0/44 (0%)	0/47 (0%)
Intermediate zone	Moderate	1/16 (6.3%)	0/23 (0%)	1/31 (3.2%)
Dangerous zone	Slow	4/10 (40%)	6/19 (31.6%)	5/8 (62.5%)

*P value < 0.001, Cut-off value for perfusion status

*Slope* (fast > 1.0 AU/sec, slow < 0.7 AU/sec), *T*_*1/2MAX*_ (fast < 10 s, slow > 18 s), *TR* time ratio (fast < 0.4, slow > 0.6)

Perfusion factors were categorized as either slow or fast-to-intermediate flow to evaluate the diagnostic value of perfusion rate as a predictive factor of anastomotic complication. Of these factors, *T*_1/2MAX_ had high sensitivity and TR had both high specificity and accuracy, suggesting that they are highly predictive of anastomotic complications (Table 5).

**Table 5:** Diagnostic values of perfusion factors for predicting anastomotic complications (*n* = 86)

	Slope	*T*_1/2MAX_	TR
Sensitivity	66.7% (4/6)	100% (6/6)	83.3% (5/6)
Specificity	92.5% (74/80)	83.7% (67/80)	96.3% (77/80)
PPV	40% (4/10)	31.6% (6/19)	62.5% (5/8)
NPV	97.4% (74/76)	100% (67/67)	98.7% (77/78)
Accuracy	90.7% (78/86)	84.9% (73/86)	95.3% (82/86)

*PPV* positive predictive value, *NPV* negative predictive value, Cut-off values (2 categories), *Slope* (slow < 0.7 AU/sec), *T*_*1/2MAX*_ (slow > 18 s), *TR* time ratio (slow > 0.6)

**Fig. 3:**
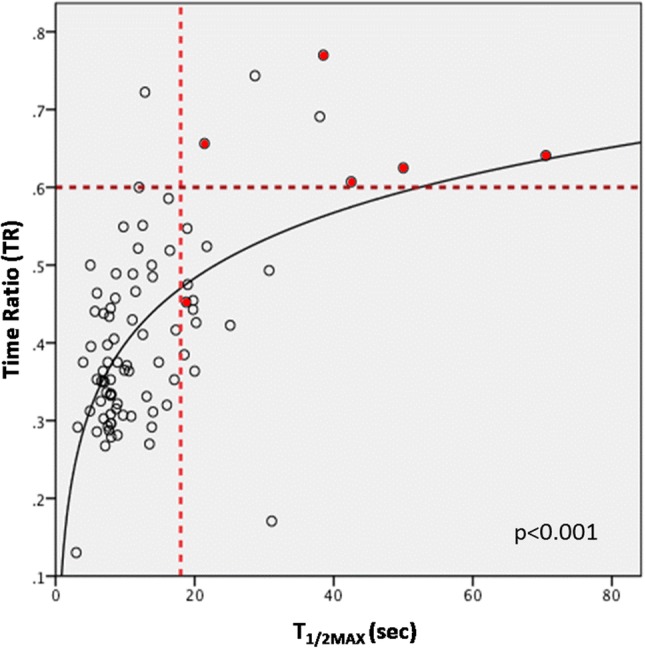
Scatter plot of *T*_1/2MAX_ and time ratio (TR) on curve estimation regression analysis with log model (*n* = 86). The cut-off value of slow *T*_1/2MAX_ (18 s) and slow TR (0.6) is indicated by a red dotted line. Patients with complications were marked with red dots

In regression analysis, the cut-off values of *T*_1/2MAX_ and TR were used to divide patients into four quadrants based on anastomotic complication occurrence (Fig. 3). When *T*_1/2MAX_ and TR were both high (i.e., slow flow), anastomotic complications occurred in 71.4% of cases. There were no anastomotic complications when *T*_1/2MAX_ and TR were both low (i.e., fast-to-moderate flow).

*T*_1/2MAX_ was associated with diabetes, advanced T status (3–4), and high ligation of IMA. Preoperative CCRTx and multiple usage of linear cutter staples (≥ 2) were associated with slow TR. Subgroup analysis of patients with low anterior resection (*n* = 55) revealed that *T*_1/2MAX_ was associated with diabetes and high ligation of the IMA. Preoperative CCRTx and SFM were also associated with low TR. The fluorescence slope was associated with smoking and SFM (Table 6).

**Table 6:** Clinical factors associated with perfusion status

Perfusion factor	Clinical factor	Total (*n* = 86)		LAR (*n* = 55)	
		Spearman’s Rho	*P* value	Spearman’s Rho	*P* value
Slope	Smoking	0.156	0.148	0.278	0.030
	SFM	− 0.109	0.314	− 0.278	0.039
*T*_1/2MAX_	Diabetes	0.284	0.008	0.332	0.014
	T status (3–4)	0.228	0.034	0.210	0.119
	IMA (high ligation)	0.237	0.028	0.292	0.030
Time ratio (TR)	Preoperative CCRTx	0.309	0.004	0.313	0.020
	linear cutter staple (≥ 2)	0.215	0.047	0.171	0.206
	SFM	− 0.168	0.119	− 0.278	0.039

*T*_1/2MAX_ time to half maximal intensity of fluorescence, *TR* time ratio (TR = *T*_1/2MAX_/*T*_MAX_), *SFM* splenic flexure mobilization, *IMA* inferior mesenteric artery, *CCRTx* concurrent chemoradiation therapy, *LAR* low anterior resection

On multivariate analysis, slow perfusion (TR > 0.6) was independent factor for anastomotic complications in a logistic regression model (OR 130.84; 95% CI 6.45–2654.75; *P* = 0.002). Anastomotic complications were significantly correlated with the novel factor TR (> 0.6) as the most reliable predictor of perfusion and anastomotic complications (Table 7).

**Table 7:** Multivariate analysis for clinical and perfusion factors associated with anastomotic complications (*n* = 86)

Clinical and perfusion factors	Odd ratio	95% CI	*P* value
Splenic flexure mobilization	0.26	0.01–7.14	0.42
Smoking	2.64	0.09–72.26	0.57
Anastomosis level (< 5 cm)	12.89	0.51–323.61	0.12
Slow TR (> 0.6)	130.84	6.45–2654.75	0.002

Multivariate analysis using Bifurcated logistic regression model

*TR* time ratio, *CI* confidence interval

## Discussion

Mesenteric vascular variations are known to cause insufficient collateral circulation in some populations. ICG imaging with suspected bowel ischemia was observed in 10.6% of rectal cancer patients in a study using robotic surgery [25]. In our previous study, when the flow of the left colic artery was blocked, the blood pressure of marginal artery was reduced by 30% or more in 11% of rectal cancer patients [26, 27]. In rectal cancer patients with anastomotic leakage, the marginal arterial stump pressure was significantly reduced [28]. These results show that unstable blood flow to left colon occurs after IMA ligation in approximately 10% of patients, which can increase the risk of anastomotic complications.

Several tests can evaluate the blood flow or vascular structure of the colon: Doppler ultrasound, laser Doppler flowmetry, angiography, and oxygen spectroscopy. These tests are not widely used in the surgical field due to the price of equipment, technical difficulties, and lack of reproducibility [23]. In particular, preoperative CT angiography can be used as a non-invasive tool to visualize the mesenteric vascular structure, but its accuracy for imaging small mesenteric arteries is limited. Recently, a near infrared system was introduced to easily perform intraoperative angiography, and it is now possible to measure blood flow in real time during robotic or laparoscopic surgery [14, 18, 29].

In previous studies, ICG fluorescence has not been clearly demonstrated to reduce anastomotic complications, but most studies have been based on the qualitative evaluation of ICG fluorescence in the colon wall or mucosa [30, 31]. It is possible that ICG angiography has not been verified as a predictor of anastomotic complications because qualitative evaluation alone is limited in accurately distinguishing changes in the microcirculation of the large intestine. Quantitative blood flow analysis is necessary to measure changes in colonic microcirculation to predict bowel viability [32]. Animal experiments using fluorescence imaging have been carried out, showing that decreased microfiltration causes intestinal necrosis [33, 34]. However, quantitative analysis of colonic perfusion is still rare in surgical practice. In recent quantitative studies, *F*_MAX_, *T*_1/2MAX_, and fluorescence slope were correlated with bowel viability or anastomotic leakage [21–23]. A delayed *T*_1/2MAX_ in graphs of jejunal perfusion reflects insufficient blood flow and may increase the possibility of postoperative ischemia [21]. In study of 112 patients in Japan, *F*_MAX_ and fluorescence slope were analyzed as predictors of anastomotic leakage, but *T*_1/2MAX_ was not a significant factor [23]. In the present study, factors related to perfusion time, such as *T*_MAX_, *T*_1/2MAX_, and TR, were shown to be significant predictors of anastomotic complications, but fluorescence intensity-related factors, like *F*_MAX_, were not statistically significant predictors. The results of quantitative analysis can be influenced not only by the perfusion state of patients but also by the characteristics of the fluorescent camera system and the video shooting conditions. We initially used a laparoscopic fluorescence camera with a xenon light source, but the brightness tended to be lower than a camera using a laser or light-emitting diode (LED). To overcome this drawback, ICG fluorescence intensity was measured using a red inversion mode with a light sky blue color after the midpoint of the study. The lighting conditions of the surgical field or the location of video shooting, either the intra-abdominal or the extra-abdominal space, can also affect the fluorescence intensity. The distance between the target colon and the laparoscopic camera lens is considered to be the most important factor causing differences in fluorescence intensity. Thus, several conditions like the fluorescence light source, color processing mode, illumination of the operating room, and the distance from the camera are all critical factors affecting the fluorescence intensity. As a result, *F*_MAX_ tended to be relatively low in the complication group, but this difference was not statistically significant. It is necessary to standardize the imaging conditions to objectively and reproducibly evaluate perfusion. By contrast, perfusion time factors, including T _MAX_, *T*_1/2MAX_, and TR, were analyzed predictors of anastomotic complications, and they showed consistent values despite the varied imaging conditions. Thus, perfusion time parameters may be highly reproducible factors for quantitative comparisons of perfusion rates.

The incidence of complications in this study was 7%, which was similar to other studies [8, 10, 35]. The initial complication rate in the year 2015 (40%, 4/10) was too high, even with ICG perfusion tests, but complications decreased rapidly in 2016 (2.8%, 1/36) and 2017 (2.5%, 1/40). The reason for the large number of complications in the initial period was that ICG angiography was performed mainly in patients whose blood flow appeared to be poor upon visual inspection. The color of the target colons seemed to reflect poor blood flow, but when ICG fluorescence was observed, it was misdiagnosed as good blood flow. Consequently, the rate of complications was elevated at the initial period of ICG fluorescence usage. The fluorescence expression of patients with complications in 2015 showed clear delays in blood flow in retrospective quantitative analysis. In fact, even if the blood flow is poor, delayed perfusion may still express fluorescence on the vessels of the colon wall, which may lead to misdiagnosis of the perfusion status by novice surgeons. Therefore, we must quantitatively track fluorescence changes in real time to establish ICG angiography as a clinically worthy tool.

In a study in Kyoto, anastomotic leakage occurred in some patients despite a change of the transection line [23]. An ischemic area may occur in the planned transection line when mesenteric fat tissue is misinterpreted as a vascularized mesentery after mesenteric division. In this situation, it is expected that short segments without fluorescence will be easily observed, and the transection line can be moved 2 ~ 3 cm to a segment with good perfusion to effectively prevent the occurrence of anastomotic complications. However, if the perfusion status is poor over 5 ~ 10 cm of the colon, the development of collateral circulation to the left colon may be insufficient, so it may be necessary to change the surgical strategy such as by avoiding anastomosis or obtaining a more extensive resection margin. In this study, 8.1% of cases required transection line changes, similar to the rate in previous studies [18, 23, 36]. Of the seven patients with transection line changes, six had no anastomotic complications, but colon necrosis occurred in one patient who underwent colorectal anastomosis with relatively visible fluorescence. However, the fluorescence intensity of the entire left colon was generally weak, and finally colonic necrosis occurred on postoperative 9th day, and emergent total colectomy was performed.

**Fig. 4:**
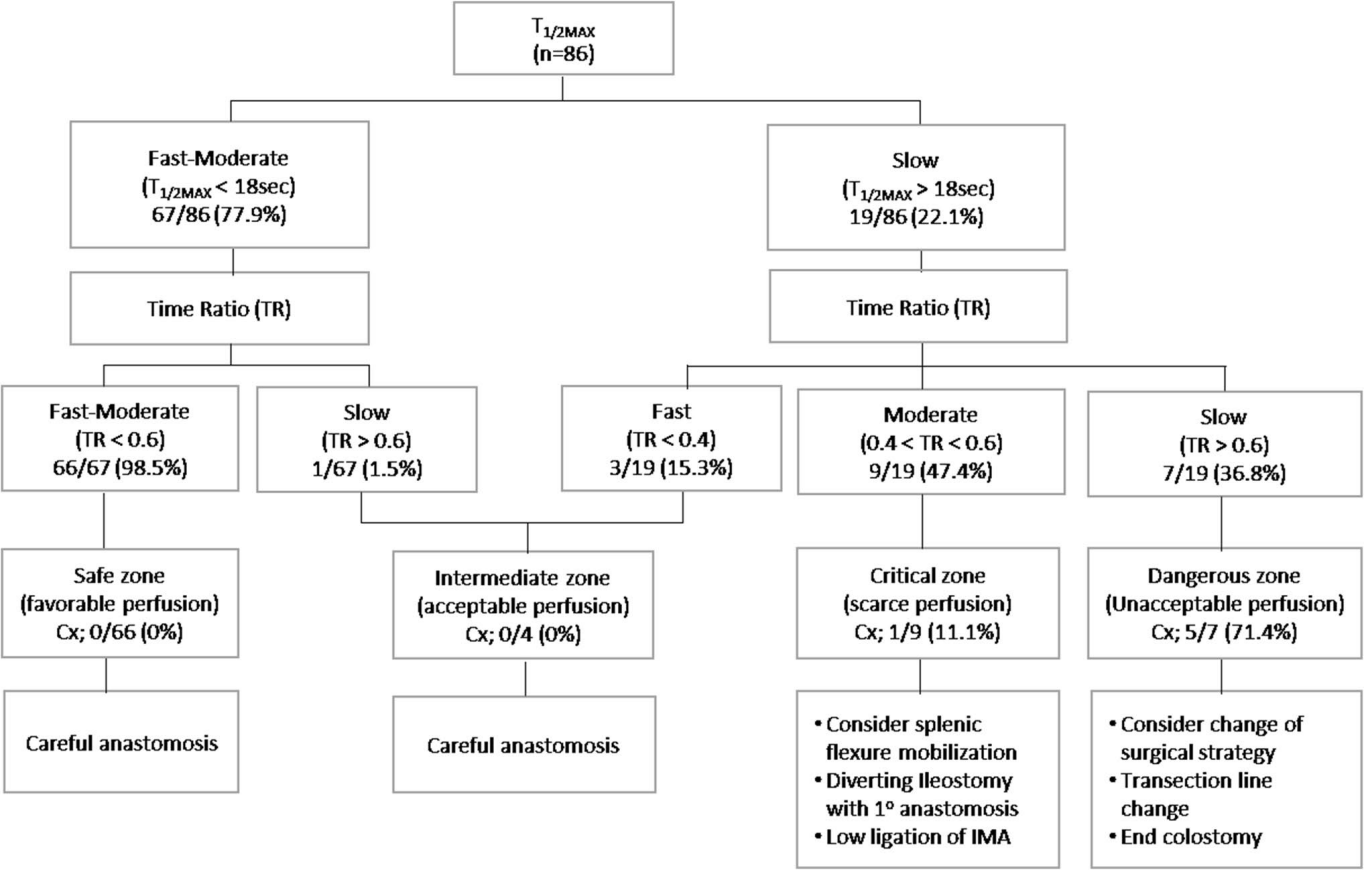
Step-by-step flow chart using perfusion factors to predict anastomotic complications after laparoscopic colorectal cancer surgery (*n* = 86). *T*_1/2MAX_ time from first fluorescence increase to half of maximum, *TR* time ratio, *Cx* anastomotic complications, Cut-off value for perfusion status, *T*_1/2MAX_ (fast < 10 s, slow > 18 s), TR (fast < 0.4, slow > 0.6)

In this study, perfusion status, reflected by *T*_1/2MAX_ and TR, could act as a predictor of anastomotic complication. As such, *T*_1/2MAX_ and TR were applied step-by-step to create a flow chart to identify poor perfusion and to classify groups by their risk of anastomotic complications. *T*_1/2MAX_, which is sensitive to slow reperfusion, was used in the first step of screening to find a risk group, and the next step was to identify individuals with poor perfusion and a high probability of anastomotic complications using TR, which has high specificity. According to their perfusion status, four risk zones were defined based on the risk of anastomotic complications. The probability of anastomotic complications in the critical region is greater than 10%, so we should actively consider splenic flexure mobilization, low ligation of the IMA, and diverting ileostomy to improve perfusion and reduce anastomotic complications. In the dangerous zone, anastomotic complications may occur in more than 70% of cases. Therefore, the colonic transection line should be moved to the proximal side, and end colostomy can be considered instead of primary anastomosis (Fig. 4).

There are several limitations to this study. First, fluorescence intensity was measured in multiple environments to identify better image conditions across 3 years. Therefore, standardization of fluorescence measurement conditions is required to find more accurate quantitative perfusion parameters. Second, protective ileostomy may be a serious confounding factor in this series, because it was performed on patients with clinical risk of anastomotic complications, such as being male, having preoperative CCRTx, LAR, or low anastomotic levels. Third, the small number of patients in our single institution is also a critical limitation. Therefore, it is difficult to prove an effect that reduces the rate of anastomotic complications via perfusion analysis, especially when considering its multifactorial nature. Thus, large-scale multicenter studies are needed to confirm that quantitative perfusion analysis has the potential to prevent anastomotic complications.

In conclusion, quantitative analysis of ICG perfusion patterns using *T*_1/2MAX_ and TR can be applied to detect segments with poor perfusion, thereby reducing anastomotic complications during laparoscopic colorectal surgery.
